# Biomarkers for Pregnancy Latency Prediction after Preterm Premature Rupture of Membranes–A Systematic Review

**DOI:** 10.3390/ijms24098027

**Published:** 2023-04-28

**Authors:** Stepan Feduniw, Michal Pruc, Michal Ciebiera, Natalia Zeber-Lubecka, Diana Massalska, Magdalena Zgliczynska, Agnieszka Pawlowska, Lukasz Szarpak

**Affiliations:** 1 Department of Gynecology, University Zürich, Frauenklinikstrasse 10, 8091 Zürich, Switzerland; 2 Research Unit, Polish Society of Disaster Medicine, 05-806 Warsaw, Poland; 3 Department of Public Health, International Academy of Ecology and Medicine, 02091 Kyiv, Ukraine; 4 Second Department of Obstetrics and Gynecology, Centre of Postgraduate Medical Education, Inflancka 6, 00-189 Warsaw, Poland; 5 Department of Gastroenterology, Hepatology and Clinical Oncology, Centre of Postgraduate Medical Education, Roentgena 5, 02-781 Warsaw, Poland; 6 Department of Genetics, Maria Sklodowska-Curie National Research Institute of Oncology, 00-001 Warsaw, Poland; 7 Department of Obstetrics, Perinatology and Neonatology, Centre of Postgraduate Medical Education, 01-813 Warsaw, Poland; 8 Student Scientific Circle of Gynecology and Obstetrics “Żelazna”, Warsaw Medical University, Żwirki i Wigury 61, 02-091 Warsaw, Poland; 9 Henry JN Taub Department of Emergency Medicine, Baylor College of Medicine, Houston, TX 77030, USA; 10 Institute of Outcomes Research, Maria Sklodowska-Curie Medical Academy, 00-136 Warsaw, Poland; 11 Research Institute, Maria Sklodowska-Curie Bialystok Oncology Center, 15-027 Bialystok, Poland

**Keywords:** PPROM, preterm premature rupture of membranes, pregnancy latency, timing to delivery, PTB, preterm birth, preterm delivery, biomarker, biomarkers, chorioamnionitis

## Abstract

Preterm premature rupture of membranes, leading to preterm birth, is associated with neonatal and maternal morbidity and mortality. The study aimed to review the existing data on the best predictive value of pregnancy latency for known biomarkers in pregnancies after preterm premature rupture of membranes. The following databases were screened for the purposes of this systematic review: Pubmed/MEDLINE, Web of Science, EMBASE, Scopus, and the Cochrane Library. The study was conducted according to the PRISMA guidelines for systematic reviews. Only a few studies assessed biomarkers predicting pregnancy duration after PPROM. IL-6, IL-8, CRP, IL1RA, s-endoglin, βhCG, AFP, PCT, urea, creatinine, oxygen radical absorbance capacity, MDA, lipocalin-2, endotoxin activity, MMP-8, MMP-9 and S100 A8/A9 were found to have a positive predictive value for delivery timing prediction. Proinflammatory biomarkers, such as IL-6 or CRP, proved to be best correlated with delivery timing, independent of the occurrence of intrauterine infection.

## 1. Introduction

Despite the crucial advances in modern perinatal medicine, the prevalence of preterm birth (PTB) has remained relatively stable over the last few decades in developed countries [1]. PTB affects 5–15% of births in developed countries and could reach 20% in developing ones [2,3]. Preterm delivery is still associated with high morbidity and mortality and may require long-term specialized medical treatment depending on the gestational age at birth [4,5].

Preterm premature rupture of membranes, also known as preterm prelabor rupture of membranes (PPROM), is one of the most common causes of PTB [6]. PPROM occurs when the amniotic membranes surrounding the fetus rupture before 37 weeks of gestation [7]. Outcomes of delivery depend on the gestational age at delivery. Delivery occurring before 22 weeks of gestation followed is called a miscarriage, as survival of delivered fetuses is rare and connected with severe morbidity. This severe morbidity and infant death rates sharply decline after the 28th week of pregnancy [8].

PPROM may complicate up to 3% of pregnancies [7,8,9,10]. Several clinical factors could help in the prediction of a high risk of PPROM, such as a history of PPROM (14%) [11] or presentations of pathogens in the vagina and cervical fluid, which could lead to intrauterine infections, and histologically diagnosed chorioamnionitis [12]. Such infections may lead to PPROM and PTB. If contractions do not start, they could lead to intrauterine fetal death and may even result in maternal sepsis and death [6]. However, intrauterine infection is more often a result of the rupture of amniotic membranes. Nevertheless, PPROM and, as a consequence, PTB could be due to increased proinflammatory cytokine levels without the presence of infection [13].

Bleeding during the first trimester of pregnancy (3–5%) could also be associated with a greater risk of PPROM [14,15]. Furthermore, socioeconomic variables, including smoking during pregnancy, predispose to the occurrence of PPROM [16,17,18].

Due to the high PPROM-related risk of PTB, the accurate prediction of pregnancy duration after its occurrence is a very important issue. Biomarkers, at this point, could be used to predict the risk of a disease or condition. Biological markers usually include proteins, hormones, or other molecules that may be measured in the maternal serum, amniotic fluid or with a urine test. These bio-indices could be used to identify women at risk of PPROM before it occurs or to evaluate the timing of pregnancy after PPROM for preventative interventions, such as antibiotic prophylaxis or steroid administration.

The study aimed to review the existing data on the best predictive value of pregnancy latency for known biomarkers in pregnancies after preterm premature rupture of membranes.

## 2. Results

**Figure 1:**
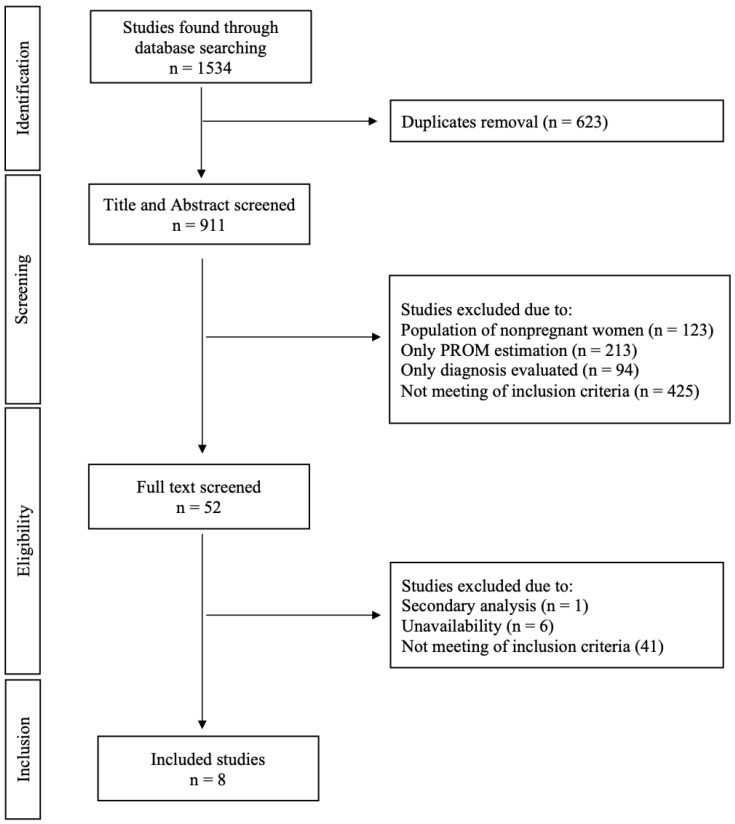
PRISMA systematic review flow diagram.

In total, 1534 articles were retrieved through the database search. This systematic review includes eight articles after removing duplicates, applying eligibility criteria, and including criteria application. A total of 553 women were assessed after PPROM, which occurred between 20 + 0 and 36 + 6 weeks of gestation. One study assessed patients with PPROM under the 20th week of gestation (17 + 4–34 + 0 weeks of gestation). Figure 1 describes a flow diagram of the detailed study selection process.

In most included studies, the diagnosis of PPROM was based on clinical assessment and a positive nitrazine test or a positive test for the presence of insulin-like growth factor-binding protein [19,20]. The predictive value of biomarkers to estimate time to delivery was calculated using receiver operating characteristic (ROC) curves based on multivariate logistic regression models and built using the clinical patient information, weeks of gestation when PPROM occurred, and the biomarker assessment. The area under the curve (AUC) was shown to establish the predictive value of each model. 

The quality of the included studies was estimated using the Newcastle–Ottawa Scale and showed that the quality of most studies was intermediate or high [21]. The results are presented in Supplementary Table S1.

Eight studies examined the predictive value of various biomarkers in patients following PPROM [22,23,24,25,26,27,28,29]. The patients were compared to those with normal pregnancies.

After the multivariate logistic regression model was built, pregnancy duration prediction models were illustrated using the Receiver Operator Characteristics (ROC). The AUC was calculated for each of the biomarkers. The AUC for delivery within 48 h and 7 days for IL-6 in the amniotic fluid were 0.871 and 0.925, respectively [27]. The AUC for s-endoglin was 0.390 [27], βhCG 0.855, AFP 0.931, prolactin 0.863 [28], and for urea, it was 0.861 [26]. Creatinine AUC was between 0.817 and 0.900, measured in the amniotic fluid [26,28]. The AUC for oxygen radical absorbance capacity was 0.800, CRP 0.673, and for MDA, it was 0.795 [25]. IL1RA reached an AUC of 0.855 [24]. The AUC for IL-8 was 0.717, lipocalin-2 0.725, MMP-9 0.755, and S100 A8/A9 0.714 [22]. Nevertheless, not all authors performed ROC analyses. Linear analysis was performed for C-reactive protein (CRP), and the odds ratio (OR) was 3 (95%CI: 1.05–11.0). The OR for malondialdehyde protein was 15 (95%CI: 1.5–162.0) [25]. A simple correlation between PTB and PPROM was shown between endotoxin activity and MMP-8 [23,29]. The results of delivery timing prediction are shown in Table 1.

The authors tried to evaluate the cut-off level of each biomarker. Only a few studies presented this result, i.e., lipocalin-2 at 0.36 μg/mL, MMP-9 at 4.27 ng/mL, S100 A8/A9 at 10.99 μg/mL, and IL-8 at 4.39 ng/mL in the amniotic fluid after transabdominal amniocentesis [22]. The cut-off values for the prediction of delivery within 48 h were >19.4 mg/dL for urea and >0.23 mg/dL for creatinine in the cervicovaginal secretions [26]. Maternal serum IL-6 cut-off levels for delivery within 48 h and 7 days were 0.70 pg/mL and 0.55 pg/mL, respectively [27]. IL1RA serum cut-off level was established at 23.8 pg/mL to predict delivery in 7 days [24]. In order to predict delivery within 3 days, CRP, malondialdehyde, and ORAC maternal serum cut-off levels were established at 0.415 mg/L, 2.085 nmol/mg and 173.71 μM/μL, respectively [25].

The levels of βhCG (r = −0.77), AFP (r = −0.77), proactin (r = −0.75), and creatinine (r = −0.68) in the vaginal fluid were negatively correlated with the duration of pregnancy [28]. Nevertheless, the cut-off levels of those proteins which could be used to predict preterm delivery were not assessed in the study.

The assessment of the included studies shows the general moderate to high quality of the studies. Most of them were prospectively collected cohorts compared with adjusted healthy pregnancies, which led to high-quality results. Nevertheless, after attempting to evaluate the correlation between biomarkers and the duration of the pregnancy, some limitations were shown. In included studies, pregnancy latency was mostly evaluated dichotomously, as preterm delivery during 3–14 days after PPROM diagnosis. Only a study by Tigga and Malik tried to evaluate the correlation of bHCG, AFP, prolactin and creatinine in the amniotic fluid with pregnancy duration after PPROM [28]. Further observation of the level of biomarkers at different stages of pregnancy could improve the quality of conclusions concerning the correlation of specific biomarkers with pregnancy duration.

The assessment of biases revealed that the lack of a comparison group of healthy pregnant women was the most common source of bias. Very important quality tools, i.e., randomization and blinding, were impossible to perform due to the diagnosis of PPROM.

## 3. Discussion

Our analysis and systematic search showed limited data on the prediction of delivery timing after PPROM. Moreover, no systematic review of biomarker value was performed. The examination of biomarkers such as leucocytosis, CRP, PCT, or proinflammatory cytokines such as IL-6 could lead to improvements in the prediction of the consequences of PPROM. Moreover, using the biomarkers combined with several clinical factors could help clinicians better estimate the delivery timing.

Cell death markers are assessed as highly predictive of preterm delivery. Nergiz Avcolu et al. demonstrated the presence of cell glycoprotein s-endoglin levels during the preterm delivery process [27]. Kim et al., Ronzoni et al., and Rahkonen et al. showed the influence of cell destruction proteins, such as lipocalin-2, MMP-8, MMP-9, and S100 A8/A9d [22,23,29]. Cell death markers could be detected as the process of membrane destruction progresses or because of the ripening of the cervix as PTB begins. A more exact evaluation of the membranes in relation to the clinical information is needed to understand the development of this process. Another factor correlated with cell destruction is oxidative stress. The biomarkers of oxidative stress, such as oxygen radical absorbance capacity or malondialdehyde, were shown by Ryu et al. to be predictive as well [25].

Gezer et al. and Tigga and Malik also reported increased amniotic fluid concentrations of urea and creatinine in preterm delivery [26,28]. This finding could be affected by fetal excess urine production as a result of fetal stress or as a reaction to the contracted uterus in the pathological process of PTB. Fetal stress as a predictor of preterm delivery after PPROM was shown to be related to PTB by Tigga and Malik, who assessed placentation biomarkers, such as βhCG or AFP [28].

Furthermore, without the diagnosis of intrauterine infection, the inflammatory biomarkers had a predictive value for delivery. Nergiz Avcıoğlu et al., Ronzoni et al., and Kim et al. assessed interleukins such as IL-6, IL-8, or their receptors, IL1RA and demonstrated their good predictive value of pregnancy duration [22,24,27]. Ryu et al., Shi et al., and Oludag et al. confirmed PCT and CRP to be good predictive markers of PTB [25,28]. A positive correlation of inflammatory proteins without the symptoms of infection is probably responsible for the reaction of cells during every delivery, especially a preterm one [13]. As mentioned before, preterm delivery (also with intact membranes and without any pathogens) involves well-known inflammatory responses in physiological delivery onset [18,30]. According to guidelines, after PPROM occurs, antibiotics prophylaxis should be given [31]. Nevertheless, there is no consensus about specific antibiotic therapy or time of antibiotics management if delivery not occurs [32]. Monitoring biomarkers could help to make a decision about the duration of antibiotic therapy. Nevertheless, further investigations should be conducted to evaluate the duration of antibiotic treatment and the correlation between biomarker levels and antibiotic dosage.

An intrauterine infection per se is a crucial factor in ensuring neonatal survival. The lack of a sufficient pregnancy duration caused by ongoing chorioamnionitis may result in fetal death or severe neonatal complications. It may also be responsible for maternal sepsis and death. Therefore, detecting an intrauterine infection as soon as possible is extremely important. In the late stages of chorioamnionitis, leucocytosis or clinical infection symptoms are frequently evident [33]. Shi et al. and Caloone et al. showed a good predictive value of CRP [34,35]. IL-6 is the most commonly described biomarker of intrauterine infection, as confirmed by Martinez-Portilla et al., Park et al., Gulati et al., Kacerovsky et al., and Cobo et al. who mentioned the best predictive value of this cytokine [36,37,38,39,40,41]. Moreover, Janku et al., Musilova et al., and Joo et al. showed pentraxin-related protein 3, also known as the TNF-inducible gene 14 protein, to be useful in chorioamnionitis prediction [42,43,44].

This analysis is not devoid of limitations. First of all, probably not every biomarker was included in the analysis because the authors are unaware of all possible proteins and cytokines involved in preterm delivery. Therefore, not every known molecule was included in the search strategy phase. Nevertheless, we tried to find novel biomarkers involved in delivery timing after PPROM. Moreover, we only assessed the predictive value of biomarkers in this analysis. Studies assessing the prediction of PPROM itself or the diagnosis of PPROM were not included. We only have estimates of whether pregnancy duration was associated with a positive or negative predictive value in terms of the biomarkers shown.

The biggest strength of this review is that this study is the first systematic analysis of known biomarkers used to predict preterm delivery or to estimate pregnancy duration after PPROM. Based on this knowledge, new prediction models could be developed to improve neonatal outcomes in pregnancies after PPROM.

The objective of the study was to evaluate the best biomarker to predict pregnancy latency. The number of included studies that assessed the influence on pregnancy duration was limited, and the information was very heterogeneous. Therefore, the evaluation of the best biomarker was difficult to perform. Nevertheless, based on the assessed biomarkers, proinflammatory biomarkers (IL-6 and CRP) seemed to be related to pregnancy duration, as inflammation is the leading cause of delivery onset in pregnancies after PPROM, even without the occurrence of intrauterine infection. Hence, the included studies do not allow such a conclusion, and further studies combining all the discussed biomarkers should be performed prospectively on larger groups of patients in order to provide researchers with more information about biomarker levels in pregnancies after PPROM.

As previously mentioned, pregnancy duration is the most important factor influencing fetal well-being. Predicting pregnancy latency is crucial to improve neonatal outcomes, whether through corticosteroid therapy, neuroprotection, or hospitalization. As pregnancies with PPROM are at a high risk of preterm delivery, the evaluation of several biomarkers is crucial. Earlier systematic reviews and meta-analyses compared the prediction of pregnancy timing mostly using clinical parameters [45,46]. Our analysis showed only a few studies taking biochemical biomarkers into consideration in their prediction models of pregnancy duration after PPROM. Moreover, the better predictive value of biomarkers was shown in the amniotic fluid after amniocentesis than in blood samples. However, a comparison between amniotic fluid levels after amniocentesis and vaginal fluid samples with leaking amniotic fluid did not show many different results. As vaginal fluid sampling is a non-invasive procedure, such an examination could be performed routinely in order to predict preterm delivery, pregnancy duration and further neonatal outcomes related to immaturity. Nevertheless, none of the studies used the potential of artificial intelligence in predicting preterm delivery. Combining the clinical variables with the described biomarkers could lead to building a high-value prediction model based on artificial intelligence [47]. The constructed model could be used in everyday clinical practice and provide the best quality of medical care for patients with PPROM. Furthermore, more research is needed to evaluate more specific biomarkers that could be used in routine medical practice to estimate the timing of delivery, potentially extend pregnancy and improve neonatal outcomes.

## 4. Methods

### 4.1. Study Design

This systematic review was conducted and written according to the Preferred Reporting Items for Systematic Reviews and Meta-Analyses (PRISMA) guidelines [48]. The checked-off list was added as Supplementary Table S2.

### 4.2. Search Strategy

The PubMed, Web of Science, Embase, and Cochrane Library databases were searched. All searches were conducted on 23 January 2023 with languages limited to English, German, or Polish. No publication time limits were imposed. The combined search strategy is presented in Table 2.

### 4.3. Inclusion Criteria

All types of evaluative study designs were included and assessed. Two reviewers (SF and MP) independently screened the studies by title and abstract. After this selection, full texts were screened. Studies that met the selection criteria were included. Every included study was assessed as 0 = not relevant, 1 = possibly relevant, or 2 = very relevant. Only publications that scored at least 1 point were included in the study. Any disagreement was discussed and resolved by the third researcher (MC).

Types of studies: Only original papers according to study design were eligible for inclusion.

Types of participants: Pregnant women with preterm premature rupture of membranes with assessed biomarkers.

Types of exposure: Preterm premature rupture of membranes and the predictive value of the biomarkers.

Types of outcome measures: Assessment of pregnancy latency and preterm delivery occurrence prediction.

Exclusion criteria: article types such as editorials, letters, conference presentations, case reports, case series, biographies, comments, editorials, preprints, lectures, newspaper articles, and other forms of popular media were excluded. Failure to meet any one of the above eligibility criteria resulted in the exclusion from the review. The third independent reviewer (MC) resolved any apparent discrepancies resulting from the selection process.

### 4.4. Data Extraction

The PICO question was, “Could biomarker levels predict pregnancy latency in pregnancies after preterm premature rupture of membranes?” Population (P): Pregnant women with preterm premature rupture of membranes and the predictive value of the biomarkers assessed. Intervention (I): Assessed predictive value of biomarkers after preterm premature rupture of membranes. Comparison (C): Pregnant women without PPROM. The outcome (O): Assessment of pregnancy latency prediction and PTB occurrence. Studies (S) included in the analyses were retrospective, prospective, or case-control ones. The PRISMA diagram was made according to the Reporting Items for Systematic Reviews and Meta-Analyses: The PRISMA Statement and presented in Figure 1 [48].

### 4.5. Quality Assessment and Risk of Bias

The risk of bias was assessed independently by two authors (SF and MP) using the Newcastle–Ottawa scale [49]. The third reviewer (MC) resolved any apparent discrepancies in the selection process. In general, the studies included were of low to moderate quality. The data are presented in Supplementary Table S1.

### 4.6. Synthesis of Results

Due to the wide variety of studies, it was impossible to perform a quantitative synthesis. Nevertheless, all prediction values of the biomarkers in the included studies were compared within the groups and presented in Table 1.

## 5. Conclusions

There are only a few studies assessing biomarkers in the prediction of pregnancy duration and intrauterine infection after PPROM. Better predictive value of biomarkers was shown in the amniotic fluid than in blood samples.

The included studies did not allow the same conclusion to predict pregnancy duration. Proinflammatory biomarkers (IL-6, CRP), even without the occurrence of intrauterine infection, seem to be related to pregnancy duration. Nevertheless, further studies combining all discussed biomarkers should be performed prospectively on larger groups of patients to provide researchers with more information about biomarker levels in pregnancies after PPROM.

## Figures and Tables

**Table 1:** Characteristics of included studies regarding the predictive value of preterm delivery or pregnancy duration.

Study	Character of The Study	Gestational Age of PPROM	Population	Biomarker	Outcomes
Kim et al. (2020) [22]	retrospective case-control study	23 + 0 to 30 + 6 weeks of gestation	88 pregnant women with PPROM who underwent amniocentesis	lipocalin-2, MMP-9, and S100 A8/A9, endostatin, Fas, IL-8, and MMP-9 in the amniotic fluid after transabdominal amniocentesis	IL-8, lipocalin-2, MMP-9, and S100 A8/A9 levels have a positive predictive value of delivery <14 days
Ronzoni et al. (2019) [23]	prospective cohort study	24 + 6 to 31 + 6 weeks of gestation	20 pregnant women with PPROM and 20 healthy pregnant women	endotoxin activity in the maternal serum	Endotoxin activity has a positive predictive value of delivery <7 days
Ronzoni et al. (2019) [24]	prospective cohort study	23 + 1 to 33 + 6 weeks of gestation	100 pregnant women with PPROM and 20 healthy pregnant women	39 cytokines in the maternal serum	IP-10, MIG, CTACK, and PDGFbb levels are related to PPROM occurrence; higher levels of anti-inflammatory cytokines in women with PPROM have a positive predictive value of delivery <7 days; IL1RA has the best negative predictive value of pregnancy duration
Ryu et al. (2017) [25]	prospective cohort study	17 + 4 to 34 + 0 weeks of gestation	72 pregnant women with PPROM	C-reactive protein (CRP), lipid peroxide, malondialdehyde, protein carbonyls, oxygen radical absorbance capacity (ORAC) in the maternal serum;	CRP, lipid peroxide levels, and low ORAC levels have a positive predictive value of delivery <3 days
Gezer et al. (2017) [26]	prospective cohort study	24 + 0 to 34 + 0 weeks of gestation	100 pregnant women with PPROM and 100 healthy pregnant women	urea and creatinine levels in cervicovaginal secretions	high levels of urea and creatinine in the vaginal fluid have a positive predictive value of pregnancy duration <48 h after PPROM; these biomarkers are also useful in PPROM diagnosis
Nergiz Avcıoğlu et al. (2015) [27]	prospective cohort study	24 + 0 to 33 + 0 weeks of gestation	55 pregnant women with PPROM and 44 healthy pregnant women	s-Endoglin, CRP and IL-6 in the maternal serum	s-Endoglin and IL-6 have a positive predictive value for pregnancy duration after PPROM, but IL-6 is more specific than s-Endoglin
Tigga and Malik (2015) [28]	case-control study	20 + 0 to 36 + 6 weeks of gestation	50 pregnant women with PPROM and 50 healthy pregnant women	βhCG, AFP, creatinine and prolactin in the vaginal fluid	AFP and creatinine are good markers to diagnose PPROM; a high level of βhCG has the best predictive value of pregnancy duration after PPROM
Rahkonen et al. (2010) [29]	prospective cohort study	22 + 6 to 36 + 6 weeks of gestation	4571 pregnant women, of which 116 had PTB and 68 had PPROM	matrix metalloproteinase 8 (MMP-8) in the cervical fluid	MMP-8 in the cervical fluid correlates with PPROM, and low levels of MMP-8 in the cervical fluid are correlated with PTB

PPROM–preterm premature rupture of membranes, CRP–c-reactive protein, MMP-8–matrix metalloproteinase 8, IL-6–interleukin 6, PTB–preterm delivery, IP-10–Interferon gamma-induced protein 10, MIG–monokine induced by gamma interferon, CTACK–cutaneous T cell-attracting chemokine, PDGFbb–Platelet-derived growth factor, subunit B, MMP-8–matrix metalloproteinase 8, MDA–malondialdehyde.

**Table 2:** Search strategy.

(Biomarker[Mesh] OR biomarker OR biomarkers OR interleukin OR procalcitonin OR CRP OR c reactive protein OR βhCG OR Human chorionic gonadotropin OR AFP OR alfa fetoprotein OR metalloproteinase OR matrix metalloproteinase) AND (preterm premature rupture of membranes OR preterm prelabor rupture of membrane OR PPROM) AND (preterm labor OR preterm delivery OR PTB OR preterm birth OR pregnancy latency OR pregnancy duration)

## Data Availability

The data supporting this study’s findings are available on request from the corresponding author (M.C.).

## Supplementary Materials

The following supporting information can be downloaded at: https://www.mdpi.com/article/10.3390/ijms24098027/s1.
