# Supplementary material for: Biomarkers for Pregnancy Latency Prediction after Preterm Premature Rupture of Membranes–A Systematic Review
Source: Int J Mol Sci. 2023 Apr 28;24(9):8027. doi: 10.3390/ijms24098027 (PMC10178250; doi:10.3390/ijms24098027)
Supplement: Supplementary file 1 [file ijms-24-08027-s001.zip › Supplementary Table S2.The Newcastle-Ottawa scale updated.pdf]

Authors	A - Selection	Exposed truly representative of average	Selection of non-exposed from the same community	Exposure of ascertained by secure record or interview	Demonstration of outcome of interest not present at the start of the study	B - Comparability	Study controls for other variables	C - Outcome	Follow up long enough for outcome to occur	Complete follow up of all subjects accounted for	Subject to follow up unlikely to introduce biases	Asssessment of outcomes	Score
Kim et al. (2020) [22]		1	1	1	1		1		1	1	1	1	9
Ronzoni et al. (2019) [23]		0	1	1	1		1		1	1	1	0	7
Ronzoni et al. (2019) [24]		1	1	1	1		0		1	1	1	1	8
Ryu et al. (2017) [25]		1	0	0	1		0		1	1	1	0	5
Gezer et al. (2017) [26]		1	1	1	1		1		1	1	1	1	9
Nergiz Avcıoğlu et al. (2015) [27]		1	1	1	1		1		1	1	1	1	9
Tigga and Malik (2015) [28]		1	1	0	1		1		1	1	1	1	8
Rahkonen et al. (2010) [29]		1	1	0	1		1		1	1	1	0	7
